# Association between levels of satisfaction with interpersonal relationships and insomnia symptoms among women working in aged-care services in Japan

**DOI:** 10.1265/ehpm.24-00399

**Published:** 2025-06-07

**Authors:** Ryuichiro Watanabe, Ai Ikeda, Hadrien Charvat, Setsuko Sato, Yuka Suzuki, Koutatsu Maruyama, Kiyohide Tomooka, Hiroo Wada, Yasunari Koyama, Takeshi Tanigawa

**Affiliations:** 1 Department of Public Health, Juntendo University Graduate School of Medicine, Tokyo, Japan; 2 Department of Health Policy and Management, Keio University School of Medicine, Tokyo, Japan; 3 Graduate School of Agriculture, Department of Bioscience, Ehime University, Ehime, Japan

**Keywords:** Interpersonal relationships, Insomnia symptoms, Work–family conflict, Structural equation model

## Abstract

**Background:**

The demand for aged-care services in Japan has surged due to the country’s aging population. Furthermore, nationwide survey on the current state of aged-care services revealed that the primary reason for the resignation of women working in these sectors was poor interpersonal relationships. Moreover, given that women working in aged-care services work in shifts around the clock to manage the health and safety of the people in their care, they are at high risk of health-related issues including insomnia symptoms. Thus, we aim to examine the association between levels of satisfaction with interpersonal relationships (LSIR) and insomnia symptoms for women working in aged-care services in Japan, as well as the effect of work–life imbalance on the association between LSIR and insomnia symptoms.

**Methods:**

In this cross-sectional study, the participants were 472 women aged 18–60 years who worked in aged-care services in Japan in 2014–2016. Insomnia symptoms were measured using the Athens Insomnia Scale, and scores of 6 or greater indicated the presence of insomnia. LSIR were assessed through self-administered questionnaires and evaluated at three levels. The association between LSIR and insomnia symptoms was evaluated using a multinominal logistic regression model. Path analysis was used to examine the potential effects of LSIR on insomnia symptoms by incorporating covariates such as work–family conflict, marital status, and depressive symptoms.

**Results:**

Compared to high LSIR, the multivariable-adjusted odds ratios (95% confidence interval) of insomnia symptoms were respectively 1.36 (0.81–2.30) and 2.42 (1.11–5.23) for medium and low LSIR. The path analysis showed that low LSIR were significantly associated with having high work-to-family (W-to-F) conflict and being single.

**Conclusions:**

Low LSIR were significantly associated with insomnia symptoms among women working in aged-care services in Japan. High W-to-F conflict exacerbated this relationship. Therefore, enhancing interpersonal relationships may be necessary for preventing insomnia. However, due to the cross-sectional nature of our study, causality cannot be inferred. Further longitudinal research is needed to better understand these associations.

## Graphical Abstract

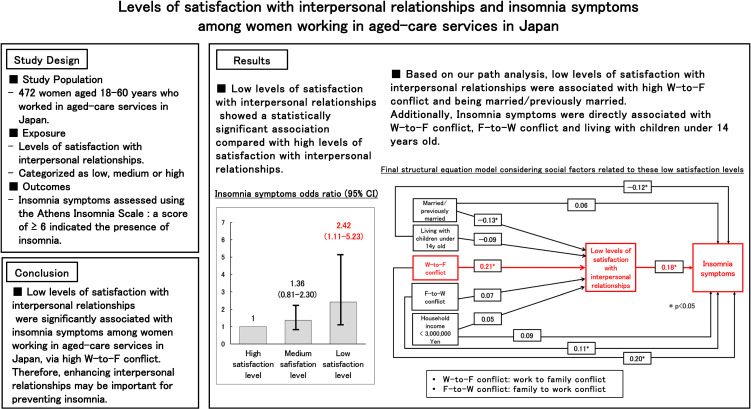

## 1. Background

Insomnia is a sleep disorder having difficulties in the initiation, maintenance, consolidation, or quality of sleep, despite sufficient time and opportunity for sleep and manifests in various forms of daytime impairment, including reduced concentration, decreased attentional capacity, emotional instability, and physical fatigue [1]. Up to 95% of Americans have reported experiencing an episode of insomnia at some point in their lives [2]. According to the Japanese Ministry of Health, Labour, and Welfare, 30–40% of Japanese adults have insomnia symptoms, and these are more common in women than men [3]. One of the causes of insomnia symptoms is stress, and people who are anxious feel stress more strongly and are therefore more likely to develop insomnia [3].

Recently, the need for aged-care services has increased in Japan due to the country’s aging population. The proportion of people aged 65 and over in the total world population was 10% in 2022 [4], while it had reached 29% (36.24 million people) in Japan in 2022 [4]. The number of annual recipients of aged-care services in Japan in 2023 was nearly double what it was in 2001 [5], and it is expected to continue increasing. The workforce in aged-care services in Japan is predominantly women [6]. Given that women working in aged-care services work in shifts around the clock to manage the health and safety of the people in their care, they are at high risk of health-related issues [7]. Furthermore, Japanese tradition dictates that women are responsible for housework and this remains deeply ingrained in Japan [8], resulting in working women tending to face work–family conflict (WFC) [9, 10]. As a result, women working in aged-care services have been reported to be affected by a range of physical and mental stresses [11–13].

Levels of satisfaction with interpersonal relationships (LSIR) are a subjective evaluation of one’s relationship with others. It is not a property of relationships, but rather a subjective experience and opinion of them [14]. Maintaining a high level of satisfaction is one of the fundamental requirements for human happiness and a sense of wellbeing [15, 16]. Some studies have reported that women experiencing work–life imbalance tend to show decreased satisfaction with interpersonal relationships [17, 18]. When individuals find it challenging to allocate sufficient time and energy to both work and their family life, they may feel stress in their relationships with both coworkers and family members. Thus, reducing causes of stress, such as work–life imbalance, is a crucial element in maintaining high LSIR. In fact, a nationwide survey of the current state of aged-care services found that the primary reason for the resignation of women working in these services was poor interpersonal relationships [19]. In addition, some studies have reported that problems in social relationships are related to sleep problems [20–22], and our previous study found that women working in aged-care services are more likely to have insomnia symptoms due to stressors such as work–life imbalance [23]. However, the associations among stressors such as work–life imbalance, LSIR, and insomnia are unclear [23, 24]. Thus, we aim to examine the association between LSIR and insomnia symptoms for women working in aged-care services in Japan, as well as the effect of work–life imbalance on the association between LSIR and insomnia symptoms.

## 2. Methods

### 2.1 Participants

This cross-sectional study included women aged 18–60 years employed in aged-care services in Japan between 2014 and 2016. Of the 745 individuals approached, 728 agreed to participate, yielding a participation rate of 97.7%. Data on work-to-family and family-to-work conflict were unavailable for 122 participants because these questions were added to a revised version of the questionnaire after data collection had begun. An additional 134 participants were excluded because they did not reside with family members. Consequently, 472 participants were eligible for analysis. Of these, five were excluded due to missing data on annual household income, five for missing data on living with children under 14 years, and one for missing data on shift work. Therefore, we analyzed data from a total of 461 women. This study was approved by the ethics review board of Juntendo University Faculty of Medicine (reference number: M20-0216) on August 4, 2014. Written informed consent was obtained from all study participants. The study was conducted in accordance with the principles of the Declaration of Helsinki.

### 2.2 Measures

#### 2.2.1 LSIR

We determined LSIR using a self-administered questionnaire from the Japanese version of the WHO QOL26 [25]. LSIR were measured via the question “How satisfied are you with your personal relationships?”, in response to which respondents chose one of five options (1 = very dissatisfied, 2 = dissatisfied, 3 = neither satisfied nor dissatisfied, 4 = satisfied, 5 = very satisfied). We placed those who responded “very dissatisfied” and “dissatisfied” in the low-LSIR group and those who responded “satisfied” and “very satisfied” in the high-LSIR group because of the small number in each of these categories. Therefore, we evaluated LSIR at three levels: 1 (low), 2 (medium [neither satisfied nor dissatisfied]), and 3 (high).

#### 2.2.2 Insomnia

Insomnia symptoms were measured using the Japanese version of the Athens Insomnia Scale, and scores of 6 or higher indicated the presence of insomnia [26].

#### 2.2.3 Work–family conflict

WFC was assessed using a self-administered questionnaire that is commonly used in Japan [27]. WFC comprises two concepts: work-to-family (W-to-F) conflict and family-to-work (F-to-W) conflict [27]. W-to-F conflict refers to the interference of work responsibilities with family life. Examples include work reducing the amount of time one can spend with family, problems at work causing irritability at home, frequent business trips keeping one away from home, and job demands draining so much energy that one struggles with demanding tasks at home. Conversely, F-to-W conflict involves disruptions at work caused by family matters, such as time spent on family issues reducing the time available for work, family worries distracting one from work, household chores affecting sleep and work performance, and family responsibilities limiting one’s relaxation or personal time. Each conflict type was measured using four questions covering three domains: time, strain, and behavior. Respondents chose one of three options (0 = never, 1 = to some extent, 2 = often) for each question, with total scores ranging from 0 to 8 for each conflict type. The total score was evaluated as low if it was 2 points or below and high if it was above 2 points.

#### 2.2.4 Covariates

The covariates were selected based on conceptual or empirical associations with insomnia symptoms and with satisfaction with interpersonal relationships reported by the previous epidemiological studies [20–22]. Depressive symptoms were considered the most important potential confounder and were evaluated accordingly using an 11-item version of the Center for Epidemiologic Studies Depression (CES-D) scale, with scores of 11 or more indicating depression [28]. Other possible confounders were age [29], alcohol status (current drinker vs. nondrinker) [30], smoking status (current smoker vs. ex-/nonsmoker) [31], occupation (clerk vs. health or care worker) [32], being a shift worker (shift vs. non-shift worker) [32], annual household income (≥3,000,000 Yen vs. <3,000,000 Yen) [33], educational attainment (high school or less vs. college or above) [33], marital status (married/previously married vs. single) [34], living with children under 14 years old (yes vs. no) [35], menopause status (menopausal vs. non-menopausal) [36], exercise habits (≥1 hour a week vs. not) [37], W-to-F conflict (high vs. low) [38], and F-to-W conflict (high vs. low) [38].

### 2.3 Statistical analysis

We compared the proportion of participants in each LSIR category to the covariates by performing general linear modelling and Cochran–Armitage tests to identify trends. The association between LSIR and insomnia symptoms was examined using multinominal logistic regression. Multivariable-adjusted models were constructed using the following cofounding factors for model 1: annual household income, marital status, living with children under 14 years old, and W-to-F conflict. For model 2, we added depressive symptoms.

Path analysis was performed to examine the potential effects of LSIR on insomnia symptoms. We used structural equation modeling [39] to assess the association between insomnia symptoms, LSIR, and social factors (marital status, living with children under 14 years old, W-to-F conflict, F-to-W conflict, and annual household income).

All statistical analyses were performed using SAS version 9.4 software (SAS Institute, Cary, NC, USA). All probability values for statistical tests were two-tailed, and values of *p* < 0.05 were regarded as statistically significant. A total of 11 participants (2.3%) had missing data of the selected covariates and were excluded from the present analysis.

## 3. Results

The basic characteristics of the participants in each of the LSIR categories are presented in Table 1. Compared to women with high LSIR, women with low LSIR were more likely to have low annual household income and be single and less likely to live with children under 14 years old. Women with low LSIR also tended to display high W-to-F conflict, depressive symptoms, and insomnia symptoms (Table 1).

**Table 1:** Demographic and prevalence ratios of insomnia symptoms according to levels of satisfaction with interpersonal relationships

		**Levels of satisfaction with interpersonal relationships**			**P**

		**High**	**Medium**	**Low**	

		**n = 142**	**n = 270**	**n = 60**	
Age	Average (SD)	39.9 (10.7)	38.0 (11.4)	37.7 (11.9)	0.25
Alcohol status, n (%)	Nondrinker	91 (64.1)	173 (64.1)	36 (60.0)	0.66
	Current drinker	51 (35.9)	97 (35.9)	24 (40.0)	
Smoking status, n (%)	Ex-/nonsmoker	119 (83.8)	214 (79.3)	45 (75.0)	0.13
	Current smoker	23 (16.2)	56 (20.7)	15 (25.0)	
Occupation, n (%)	Clerk	17 (12.0)	33 (12.2)	12 (20.0)	0.21
	Health/care worker	125 (88.0)	237 (87.8)	48 (80.0)	
Shiftwork, n (%)	Non-shift	103 (72.5)	188 (69.9)	42 (70.0)	0.63
	Shift	39 (27.5)	81 (30.1)	10 (30.0)	
Annual household income, n (%)	<3,000,000 Yen	16 (11.3)	40 (15.1)	14 (23.3)	0.04
	≥3,000,000 Yen	126 (88.7)	225 (84.9)	46 (76.7)	
Educational attainment, n (%)	High school or less	50 (35.2)	76 (28.2)	27 (45.0)	0.57
	College or above	92 (64.8)	193 (71.8)	33 (55.0)	
Marital status, n (%)	Married/previously married	108 (76.1)	183 (67.8)	33 (55.0)	0.003
	Single	34 (23.9)	87 (32.2)	27 (45.0)	
Living with children under 14 years old, n (%)	No	87 (61.3)	174 (65.4)	45 (77.6)	0.04
	Yes	55 (38.7)	92 (34.6)	13 (22.4)	
Menopause status, n (%)	Not menopausal	115 (81.0)	218 (81.0)	48 (80.0)	0.90
	Menopausal	27 (19.0)	51 (19.0)	12 (20.0)	
Exercise habit, n (%)	No	113 (79.6)	206 (76.3)	43 (71.7)	0.22
	≥1 hour a week	29 (20.4)	64 (23.7)	17 (28.3)	
W-to-F conflict, n (%)	Low	95 (66.9)	158 (58.5)	17 (28.3)	<0.001
	High	47 (33.1)	112 (41.5)	43 (71.7)	
F-to-W conflict, n (%)	Low	93 (65.5)	172 (63.7)	32 (53.3)	0.16
	High	49 (34.5)	98 (36.3)	28 (46.7)	
Depressive symptoms, n (%)	No	131 (92.9)	215 (79.6)	26 (43.3)	<0.001
	Yes	10 (7.1)	55 (20.4)	34 (56.7)	
Insomnia symptoms, n (%)	No	114 (80.3)	188 (69.6)	25 (41.7)	<0.001
	Yes	28 (19.7)	82 (30.4)	35 (58.3)	

A general linear model was used to assess the association with age. The Cochran–Armitage test for trend was used to assess for associations with alcohol status, smoking status, occupation, shiftwork, annual household income, educational attainment, marital status, living with children under 14 years old, menopause status, exercise habit, W-to-F conflict, F-to-W conflict, depressive symptoms, and insomnia symptoms.

F-to-W: family to work, SD: standard deviation, W-to-F: work to family.

Depressive symptoms are defined as a CES-D score ≥11. Insomnia symptoms are defined as an AIS score ≥6. High W-to-F conflict and high F-to-W conflict are defined according to the National Survey of Midline Development in the United States as a total score for each metric of ≥3.

**Fig. 1:**
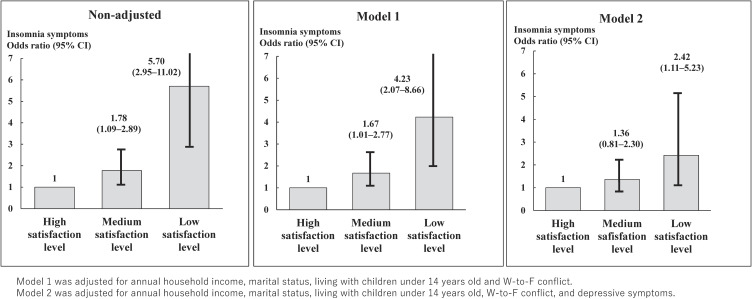
Multivariable-adjusted odds ratios between insomnia symptoms and satisfaction with interpersonal relationships estimated by multinomial logistic regression.

After adjusting for the relevant confounders in model 1 (Fig. 1), we found statistically significant associations between high and medium LSIR and between the high and low satisfaction levels, respectively. The multivariable-adjusted odds ratios (95% confidence interval [CI]) for having insomnia symptoms were 1.67 [1.01–2.77] for medium LSIR and 4.23 [2.07–8.66] for low LSIR compared to high LSIR. After adjusting further for depressive symptoms in model 2 (Fig. 1), because our previous study found that depressive symptoms were a strong cofounder [23], the association for medium LSIR was no longer statistically significant (multivariable-adjusted odds ratio [95% CI]: 1.36 [0.81–2.30]), but the association for low LSIR remained significant (2.42 [1.11–5.23]). The standard errors (SEs) of all explanatory variables in the logistic regression model were within an appropriate range, each exceeding 1.0. Therefore, the influence of multicollinearity was considered negligible.

**Fig. 2:**
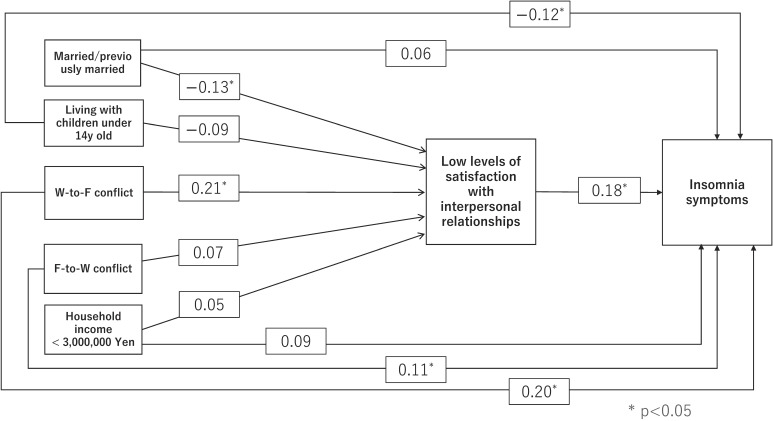
The associations between insomnia symptoms, low satisfaction level and social factors based on the structural equation model.

Because the logistic regression (Fig. 1) demonstrated the association between satisfaction with interpersonal relationships and insomnia symptoms, we selected social factors that could potentially relate to the daily work-life balance of women working in aged-care services. We conducted an exploratory analysis to investigate how these factors might be associated with the relationship between satisfaction with interpersonal relationships and insomnia symptoms, using a path model as the analytical framework. The path analysis (Fig. 2) shows the effects of the related social factors on the association between low LSIR and insomnia symptoms, based on our path analysis. We found that low LSIR were associated with high W-to-F conflict (*β* = 0.21) and being married/previously married (*β* = −0.13). Additionally, we found that Insomnia symptoms were directly associated with W-to-F conflict (*β* = 0.20), F-to-W conflict (*β* = 0.11) and living with children under 14 years old (*β* = −0.12).

## 4. Discussion

This is the first study to examine the association between LSIR and insomnia symptoms among women working in aged-care services in Japan, as well as the effect of work–life imbalance on the association between LSIR and insomnia symptoms for women working in aged-care services in Japan. We found that women with low LSIR were more likely to have insomnia symptoms, have high W-to-F conflict, and be single.

Our results showed similarities to the findings of previous studies [20–22]. Masoudnia (2015) reported that employees with deficient or poor relationships with others suffered from daytime dysfunction caused by insomnia or poor sleep quality [22]. In addition, Kent et al. (2015) reported that poor relationships were related to lower sleep quality [21]. Therefore, emotional vulnerability may be a possible mediating factor, leading to increased vigilance and reduced sleep quality [22]. Moreover, in the present study, we evaluated the effect of depressive symptoms on the association between LSIR and insomnia. Depressive symptoms may strongly influence the risk of developing insomnia symptoms [40–42]. However, our study found that the association between low LSIR and insomnia symptoms remained statistically significant after adjusting for depression. This suggests that there might be other factors influencing the association between LSIR and insomnia symptoms.

However, we also found some differences from previous studies. We examined the effect of work–life imbalance on the association between LSIR and insomnia symptoms using path analysis and showed that low LSIR was significantly associated with high W-to-F conflict. The path analysis suggested that among women working in aged-care services, chronic stress related to insomnia symptoms may be involved in the association between low satisfaction with LSIR and high W-to-F conflict. This situation could trigger two possible biological mechanisms. First, the chronic stress resulting from low LSIR and high W-to-F conflict may stimulate the release of stress hormones including cortisol [43]. Increased cortisol levels have been reported to be associated with chronic insomnia [44]. Second, stress also stimulates the sympathetic nervous system, keeping it active [45]. Therefore, stimulation of the brain and overactivation of the sympathetic nervous system through perceived stress may result in insomnia symptoms.

A strength of our study was the adequate sample size (more than 400 subjects). This was also the first study to examine the association between LSIR and insomnia by incorporating social environmental factors. However, the study has several limitations. First, the questionnaire regarding LSIR consisted of a single question. Thus, the information provided limited our evaluation of the participants’ satisfaction levels. Second, the participants in this study were women working in aged-care services, and a relatively high proportion of them were the night shift. Thus, generalizability to other populations, such as women working outside the aged-care service sector, may be limited, and the findings may not be applicable to the broader working population. However, the results are considered applicable to women employed in hospitals and aged-care services. Third, since this study was cross-sectional, we could not determine any causal relationship between LSIR and insomnia symptoms. Additionally, other unmeasured confounders and the possibility of reverse causation may still influence the results. Therefore, a longitudinal study should be conducted to determine the direct effect of LSIR on developing insomnia. Lastly, unmeasured confounding factors like emotional symptoms (e.g., anxiety, anger, and sadness) [46] and mindfulness in relationships (e.g., self-awareness, gratitude, and open communication) [47] may influence the relationship between LSIR and insomnia symptoms [20].

## 5. Conclusions

Low LSIR were significantly associated with insomnia symptoms among women working in aged-care services in Japan, via high W-to-F conflict. Therefore, enhancing interpersonal relationships may be important for preventing insomnia.

## Abbreviations

LSIR: Levels of satisfaction with interpersonal relationships
WFC: work–family conflict
W-to-F: work-to-family
F-to-W: family-to-work
WHO QOL26: World Health Organization Quality of Life 26
CES-D scale: Center for Epidemiologic Studies Depression scale

## Declarations

### Data availability

Data cannot be shared for privacy or ethical reasons.
